# Treatment Planning Methods for Dose Painting by Numbers Treatment in Gamma Knife Radiosurgery

**DOI:** 10.1016/j.adro.2024.101534

**Published:** 2024-05-01

**Authors:** Benjamin Z. Tham, Dionne M. Aleman, Håkan Nordström, Nelly Nygren, Catherine Coolens

**Affiliations:** a Department of Radiation Oncology, Princess Margaret Cancer Centre, Toronto, Ontario, Canada; b Department of Mechanical and Industrial Engineering, University of Toronto, Toronto, Ontario, Canada; c Elekta Instrument AB, Stockholm, Sweden

## Abstract

**Purpose:**

Dose painting radiation therapy delivers a nonuniform dose to tumors to account for heterogeneous radiosensitivity. With recent and ongoing development of Gamma Knife machines making large-volume brain tumor treatments more practical, it is increasingly feasible to deliver dose painting treatments. The increased prescription complexity means automated treatment planning is greatly beneficial, and the impact of dose painting on stereotactic radiosurgery (SRS) plan quality has not yet been studied. This research investigates the plan quality achievable for Gamma Knife SRS dose painting treatments when using optimization techniques and automated isocenter placement in treatment planning.

**Methods and Materials:**

Dose painting prescription functions with varying parameters were applied to convert voxel image intensities to prescriptions for 10 sample cases. To study achievable plan quality and optimization, clinically placed isocenters were used with each dose painting prescription and optimized using a semi-infinite linear programming formulation. To study automated isocenter placement, a grassfire sphere-packing algorithm and a clinically available Leksell gamma plan isocenter fill algorithm were used. Plan quality for each optimized treatment plan was measured with dose painting SRS metrics.

**Results:**

Optimization can be used to find high quality dose painting plans, and plan quality is affected by the dose painting prescription method. Polynomial function prescriptions show more achievable plan quality than sigmoid function prescriptions even with high mean dose boost. Automated isocenter placement is shown as a feasible method for dose painting SRS treatment, and increasing the number of isocenters improves plan quality. The computational solve time for optimization is within 5 minutes in most cases, which is suitable for clinical planning.

**Conclusions:**

The impact of dose painting prescription method on achievable plan quality is quantified in this study. Optimization and automated isocenter placement are shown as possible treatment planning methods to obtain high quality plans.

## Introduction

It is well-established that several tumor types have heterogeneous radiation sensitivity, such as from varying levels of hypoxia or cell density, and the dose painting treatment method, which delivers a nonuniform dose to the target, may be used to improve patient outcomes.^1^, ^2^, ^3^, ^4^ One method of dose painting is adaptive dose painting, where the prescription is adjusted partway through the treatment process based on the patient's response during previous treatment sessions.^5^^,^^6^ The second is subvolume boosting, sometimes referred to as “dose escalation,” “dose sculpting,” or “dose painting by contours,” where 1 or more volumes within a target are prescribed a higher dose.^4^^,^^7^, ^8^, ^9^ A third method is dose painting by numbers (DPBN), where each voxel's prescription is adjusted based on image intensities, typically from positron emission tomography (PET) or magnetic resonance imaging (MRI), which is conceptually similar to subvolume boosting but with voxel-level adjustments.^2^^,^^10^

With the increasing precision and accuracy of medical imaging and treatment, voxel-level dose prescriptions in DPBN have been demonstrated as clinically feasible and beneficial by numerous studies.^6^^,^^10^, ^11^, ^12^, ^13^, ^14^, ^15^, ^16^, ^17^ Furthermore, geometric uncertainties in DPBN have been addressed through robust optimization^18^, ^19^, ^20^ and geometric expansion.^21^ The precision required for DPBN, together with the inherent precision of stereotactic radiosurgery (SRS), makes their combination interesting to study, where the precision in SRS potentially makes DPBN more achievable and DPBN expands the possibility of normal tissue sparing already present in SRS.

SRS treatment delivers high radiation doses in 1 or a few high-precision fractions to small tumors in the brain, and the ongoing development of SRS systems increases the feasibility of dose painting SRS treatment. Most Gamma Knife ICON and Elekta Esprit machines, for example, allow immobilization with a thermoplastic mask and high definition motion management, so SRS treatments with larger target volumes and multiple fractions (also called “stereotactic radiation therapy”) are more practical and prevalent.^22^, ^23^, ^24^ With the ability to treat larger tumors with the Gamma Knife systems, the spatial dose variation of dose painting is more achievable, and the established benefits of dose painting can thus be extended to SRS and stereotactic radiation therapy.

Gamma Knife plans with standard single prescriptions are typically manually planned, also known as “forward planning,” through a combination of clinician experience and trial and error. The relative complexity of DPBN prescriptions, however, with up to hundreds or thousands of individual voxel prescriptions, means that automated planning, which includes inverse planning optimization and isocenter placement, is greatly beneficial in finding optimal treatment plans from the given prescription.

Optimization in Gamma Knife systems has been considered in several studies. For the previous Gamma Knife systems that used several collimator helmets, different optimization methods have been studied, including a multistep process with mixed integer programming,^25^^,^^26^ nonlinear programming method with migrating isocenters^27^ or isocenters placed along a target's medial axis transform,^28^ and a combination of packing spheres along a medial axis transform then linear optimization for radiation time.^29^

The newer Gamma Knife Perfexion and ICON systems (Elekta) have movable sources in 8 independent sectors, allowing complex dose distribution profiles by using combinations of 3 collimator sizes from the 8 sectors. This flexibility leads to quite a challenging optimization problem for which a few optimization methods have been developed. Ghobadi et al^30^^,^^31^ use a hybrid grassfire and sphere-packing (GSP) algorithm to find isocenter locations, adapted from a method proposed by Wagner et al^32^ for linear accelerator SRS, followed by a sector-duration optimization (SDO) method to find optimal collimator sizes and radiation times. Ghaffari et al^33^ extend the SDO method to consider multiple methods for beam-on time (BOT) penalties, and Cevik et al^34^ implement SDO combined with isocenter selection from a given set. Levivier et al^35^ use a convex optimization approach to simultaneously find isocenter locations and radiation times. Oskoorouchi et al^36^ use a semi-infinite linear programming method and predetermined isocenters. Sjölund et al^37^ apply a linear programming method using predetermined isocenters, and this is the method the current clinical optimization software (Lightning) is built on.

Besides a suggestion of dose painting SRS possibility by Sjölund et al,^37^ the consideration of the impact of dose painting on SRS optimization has been minimally studied. This study attempts to start filling the gap in 3 ways, by (1) examining the achievable quality of dose painting Gamma Knife plans through optimization, (2) quantifying how different prescription methods would affect plan quality, and (3) exploring how automated isocenter placement would affect dose painting Gamma Knife treatment plan quality.

## Methods and Materials

### Prescription functions

A selection of 10 cases, previously treated in an Research Ethics Board-approved study at Princess Margaret Cancer Centre, were selected to provide a range of target volume and number. These cases were treated with a Gamma Knife ICON machine, with 1 to 8 targets of total volumes from 0.11 to 26 cc and clinical single prescriptions from 12 to 24 Gy. Apparent diffusion coefficient (ADC) MRI images were used as an indicator of cell density heterogeneity.^38^^,^^39^ Cell density has been shown to impact dose sensitivity^40^, ^41^, ^42^ and would thus affect the desired dose prescribed in dose painting. Although ADC images can be noisy, future studies may address the use of noisy images with lowered dose variation in DPBN prescription or make use of higher-resolution imaging available through MRI^13^ or PET.^6^^,^^11^^,^^12^^,^^15^^,^^16^ The foci of the study are treatment planning methods, so ADC images are suitable as an illustrative example to generate heterogeneous dose painting prescriptions. In this study, lower ADC image intensities are used as an indication of high tumor cell density,^38^^,^^39^ so those voxels were prescribed higher doses.

Using the image intensities, dose painting prescriptions are found for each voxel through applying a prescription function. This study uses prescription functions adapted from Bowen et al.^43^ One prescription function is polynomial and the other sigmoid, each having different advantages. Polynomial prescription functions have simpler parameterization and have been used in several earlier dose painting studies with linear functions,^17^^,^^44^^,^^45^ whereas sigmoid functions mimic biologic dose-response functions.^43^

The integral boost method we use is similar to prescription methods that use dose redistribution,^46^^,^^47^ with the mean prescription kept fixed. Compared with maximum boost methods, integral boost methods have the advantage of allowing comparisons to a control case where a uniform boost is applied such that the single uniform prescription is equal to the integral boost mean prescription.^5^

A change this paper introduces is using relative image intensities instead of absolute image intensities. With absolute intensity, the set minimum prescription will not be given for a voxel unless it has 0 image intensity, which may be possible with Cu-ATSM (copper-diacetyl-bis(N-methylthiosemicarbazone)) PET imaging used by Bowen et al^43^ but cannot be generalized to all types of images. Using relative intensity, as proposed with some studies with linear prescription functions,^5^^,^^44^^,^^45^ allows that generalization and ensures there are voxels given the minimum prescription. As the relationship between image intensity, tumor biology, and prescription dose is complex,^5^^,^^43^ this change means we do not assume that our image intensities are directly indicative of hypoxia or radiosensitivity. For example, if the lowest numerical intensity in our target image is 250 and the set minimum prescription is 18 Gy, using absolute intensity in calculations would have no voxels assigned in 18 Gy prescriptions, whereas using relative intensity would.

In this study, the prescription functions for voxel *i* (*i* = 1*,…,N*) are

$$d_{i}=D_{\text{min}}+d_{\text{boost}}$$

$$d_{\text{boost}}=\left\{ \begin{array}{c} \Delta D_{\text{mean}}\cdot \frac{{\left(h_{i}-h_{\text{min}}\right)}^{n}}{\langle {\left(h-h_{\text{min}}\right)}^{n}\rangle }\;\left(polynomial\;case\right)\\ \Delta D_{\text{mean}}\cdot \frac{\text{exp}\left\{-\text{exp}\left[me\frac{h_{i}-h_{c}}{\langle h-h_{\text{min}}\rangle }\right]\right\}}{\sum_{i=\;1}^{N}\text{exp}\left\{-\text{exp}\left[me\frac{h_{i}-h_{c}}{\langle h-h_{\text{min}}\rangle }\right]\right\}}\;\left(sigmoid\;case\right) \end{array}\right.$$

where *d_i_* is the prescribed dose for voxel *i* with heterogeneity indication of *h_i_,* which is equal to relative voxel image intensity for this study, and *d*_boost_ is the voxel's boost above the minimum prescribed dose. *h*_max_, *h*_min_, and *h_c_* are the maximum, minimum, and sigmoid center of *h_i_* values. *D*_min_ and ∆*D*_mean_ are prescribed minimum dose and mean integral dose boost. Polynomial function has a voxel's prescription adjusted by its imaging intensity relative to the minimum intensity (*h_i_* − *h*_min_) and exponent *n,* with a suitable denominator for ensuring mean integral boost is achieved. The sigmoid function uses a combination of exponential functions to achieve a sigmoid curve in boost distributions, with sigmoid center *h_c_* set at the mean of the maximum and minimum voxel intensities to emphasize the difference from polynomial functions. *m* is the sigmoid slope, determined at the sigmoid center, and *e* is Euler's number. The denominator for the sigmoid function similarly ensures the mean integral boost is achieved.

To generate a variety of prescription functions, parameters are varied, with polynomial exponent *n* = {0*.*5*,*1*,*2}, sigmoid slope *m* = {2*,*4*,*10}, and integral mean boost = {5%*,*10%*,*15%*,*20%*,*25%}, with percentages relative to *D*_min_. With these prescription functions, various prescription dose distributions are created for each case, and respective Gamma Knife treatment plans are found through optimization.

### Optimization

The optimization method is adapted from a semi-infinite linear programming formulation,^36^^,^^48^ with modifications to account for dose painting workflow and prescriptions. The original formulation uses quadratic overdose and underdose penalties approximated with linear equations and is solved with an interior-point constraint generation algorithm previously shown to be efficient in producing Gamma Knife treatment plans.^36^

Besides the change of single prescription to individual voxel prescriptions, 3 additional modifications were made to the formulation. First, we add a linear beam-on time (BOT) penalty, which in preliminary analysis reduced BOT while not significantly impacting plan quality. Second, a target overdose penalty threshold is set at 200% of the prescription dose, which is analogous to single prescription cases with a common 50% prescription isodose, meaning that the optimization enforces planning to be close to the 50% isodose level. Third, we employ 2 sets of normal tissue overdose penalties; 1 to lower overall normal tissue dose and 1 to improve dose conformity for the normal tissue immediately surrounding the target.

Optimization parameters are preset depending on target volume and not tuned for each case. Although this is not reflective of the clinical planning scenario where each case and prescription will have tuned parameters to get the best possible plan, using consistent parameters improves comparability and allows the demonstration that optimization can be used to create acceptable plans even before tuning.

### Isocenter placement

We investigate obtaining isocenter locations through a modified grassfire sphere-packing (GSP) algorithm. As comparison, we use isocenters previously placed in clinical planning and the isocenters generated by the Leksell gamma plan (LGP) isocenter fill algorithm, currently available as a feature of the Gamma Knife Lightning treatment planning system and used with default parameters.

The modified GSP algorithm^30^^,^^31^ packs spheres into the target volume by geometry, and the centers of these spheres are used as isocenters. We modify the algorithm to remove the restriction of sphere sizes to the current 3 Gamma Knife collimator sizes, which prioritizes large sphere placement near the target center and smaller spheres at the boundaries, and to instead use uniformly sized spheres. Although dose distributions need not be spherical when combining collimators from different sectors, describing them as spheres serves as a suitable proxy in finding isocenter locations. As the prescription complexity increases with dose painting, increasing the number of isocenters is likely to increase treatment plan quality by giving more degrees of freedom. To investigate this, the GSP algorithm is applied with various numbers of isocenters in the optimization, starting with the same number of isocenters as the clinical plan and increasing from there.

### Plan evaluation

The created treatment plans are scored on SRS-relevant metrics modified for dose painting: coverage, selectivity, Paddick conformity index (PCI), and efficiency index (EI). For this study, overall conformity is measured with a modified PCI, calculated from a product of coverage and selectivity, and overall normal tissue sparing is estimated with a modified EI. Further investigation and rationale for the metrics chosen can be found in other studies,^49^ but the modified metrics used here are summarized as follows.

Coverage^∗^ is calculated relative to individual voxel prescriptions instead of a uniform single prescription. Selectivity_mean_ and EI_50%_*_,_*_mean_ are modified to use the mean prescribed dose instead of a set single prescribed dose. PCI^∗^_mean_ uses a product of coverage^∗^ and selectivity_mean_, as PCI may be calculated as a product of coverage and selectivity. For this paper, the use of “*” indicates the use of a modified metric to account for variable prescriptions.

The equations for metrics used in this paper are

$$\text{Coverag}e^{*}=\frac{\sum \text{voxel}s_{\text{planned}\;\text{dose}\;\geq \;\text{prescribed}\;\text{dose}}}{\text{GTV}}$$

$$\text{Selectivit}y_{\text{mean}}=\frac{\text{TT}V_{\text{mean}}}{\text{PI}V_{\text{mean}}}$$

$${\text{PCI}}_{\text{mean}}^{*}=\text{Coverag}e^{*}\times \text{Selectivit}y_{\text{mean}}$$

$$EI_{50\%,\text{mean}}=\frac{\text{Integral}\;\text{Dose}\;\text{of}\;\text{GTV}}{\text{Integral}\;\text{Dose}\;\text{of}\;\text{PI}V_{50\%,\text{mean}}}$$

where GTV is gross tumor or target volume, TTV_mean_ is treated target volume or target volume covered by the mean prescription isodose line, and PIV_X_ is the prescription isodose volume or volume covered by the X prescription isodose line (mean, or 50% of mean). For this paper, overall conformity is estimated with PCI^∗^, and overall normal tissue sparing is estimated with EI_50%_.

Target dose is further studied with quality-volume histograms (QVH),^44^ a tool commonly used in dose painting radiation therapy studies where volume is plotted against quality (or relative dose) instead of absolute dose. Additionally, the dose painting plans are compared with optimized single prescription plans: a uniform boost is applied equal to the mean boost, so the single prescription plan would have the same mean prescription as the integral boost dose painting prescriptions.

## Results

### Dose painting plan quality

**Figure 1:**
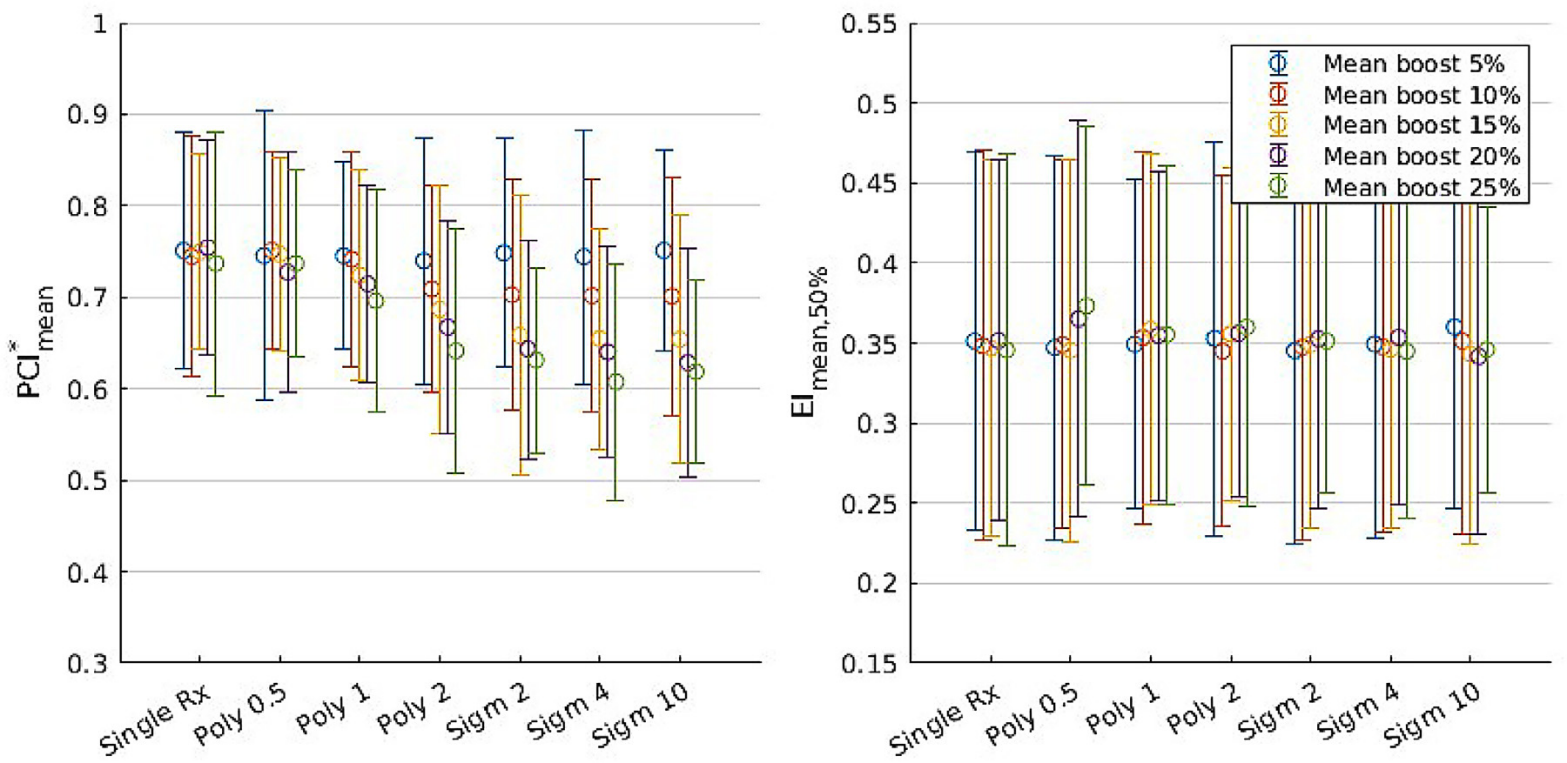
Paddick conformity index and efficiency index for dose painting plans with prescriptions using the integral boost method, with error bars indicating SD. Data are grouped by prescription function and by the level of boost, then averaged over all 10 cases. Single-prescription plans that use a uniform mean boost across all voxels, analogous to current stereotactic radiosurgery treatments with single prescriptions, are provided as a comparison.

Figure 1 shows the impact of integral boost dose painting on plan quality. Compared with single-prescription plans, where the boost applied is uniform, mean boost of 5% does not negatively impact PCI^∗^_mean_ and EI_mean,50%_. There is a small impact on PCI^∗^_mean_ when using a polynomial function with exponent 0.5 or 1, and the largest impact is with using sigmoid functions. The error bars are large, as the large variety of case features results in a large spread of metric values. However, the mean values demonstrate qualitatively that dose painting method affects conformity, as measured by PCI^∗^_mean_: conformity remains consistent regardless of prescription form when mean boost is kept at 5%, and for some prescription forms (polynomial form with exponent 2, all sigmoid form options), conformity decreases as mean boost is increased to 25%. EI_mean,50%_ remains mostly unchanged through prescription function and boost level, indicating that dose painting plans may be expected to have comparable normal tissue sparing when judging from 50% isodose of mean prescription.

**Figure 2:**
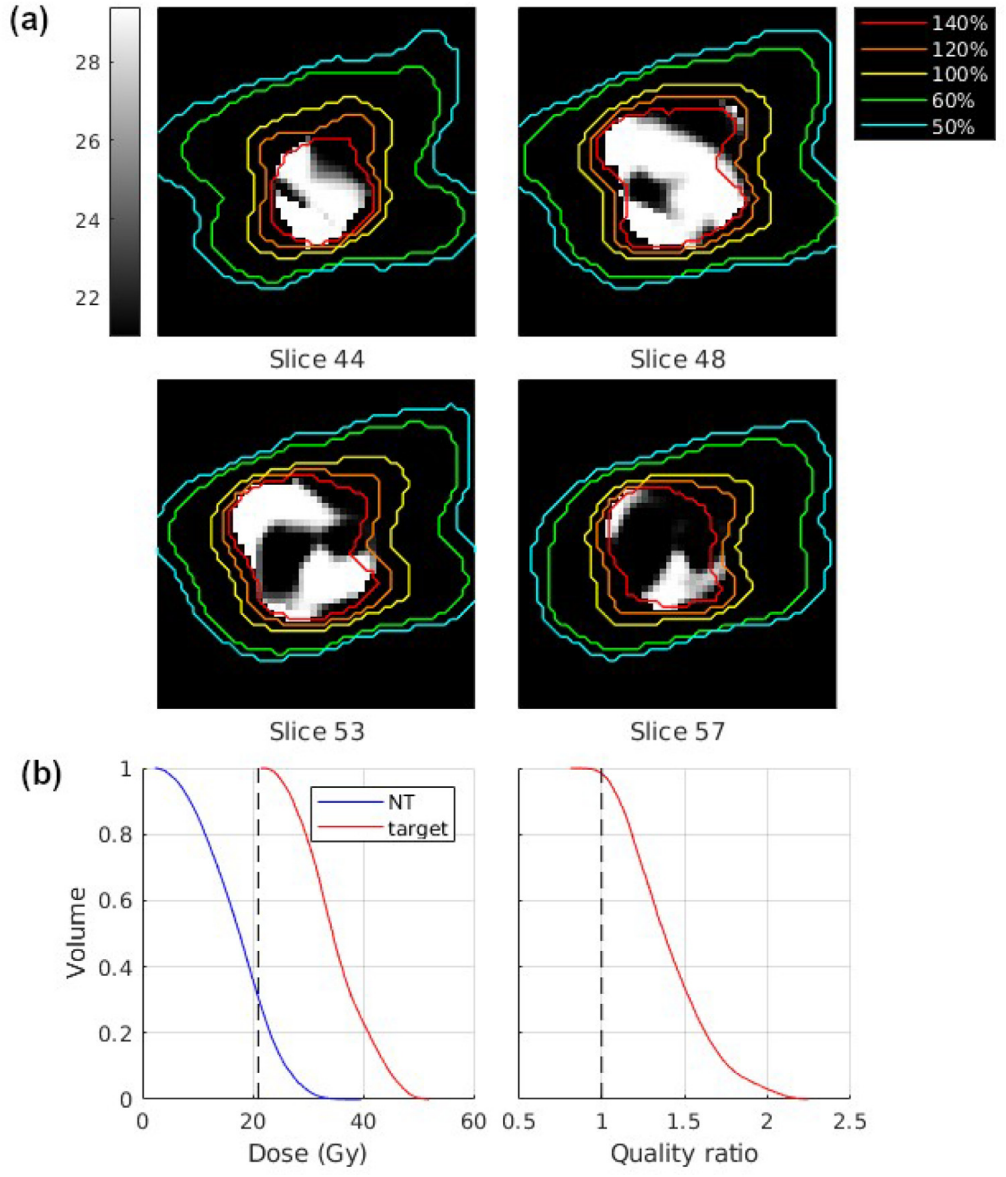
Sample dose painting plan using a sigmoid prescription function with slope 2, minimum prescription 21 Gy, and mean boost of 20% (4.2 Gy). (a) Slices show the individual voxels’ dose painting prescriptions, between 21 and 29.4 Gy, and isodose lines show the planned dose from the optimized treatment plan, relative to 21 Gy. (b) Dose-volume histogram and quality-volume histograms are shown for the target and dose-volume histogram for the normal tissue surrounding the target used in plan optimization.

An example integral boost dose painting plan, using a sigmoid prescription function, is shown in Fig. 2. The image slices show the prescribed dose in Gy, and the isodose lines show that the planned dose has a degree of sculpting, matching higher planned dose regions to higher prescribed dose regions. The regions of high- and low-dose prescriptions are pronounced with sigmoid functions, even with a smaller sigmoid slope of 2. This target has coverage^∗^ 0.98, which can be read from the QVH, selectivity_mean_ 0.73. The target's dose-volume histogram shows all voxels having planned dose above the minimum prescription of 21 Gy, but when scaled by individual prescriptions, the QVH shape is similar to what one may expect with single prescription plans.

Table 1 shows a breakdown of metrics by integral boost level, prescription function, and case. Increased integral boost reducing PCI and EI, increasing BOT, and case features are similarly strong factors in determining metrics. Sigmoid functions have lower selectivity and PCI compared with polynomial functions, but EI is not affected much by prescription function form.

**Table 1:** Plan metrics scores for integral boost dose painting plans, divided by boost level, by prescription function form and by case

	Data set	Cov.*	Sel._mean_	PCI*_mean_	GI_mean_	EI_mean,50%_	BOT (min)
	Clinical, single prescr.	0.947	0.701	0.662	3.21	0.362	111.3
Boost level	Integral boost 5%	0.962	0.775	0.746	3.86	0.351	134.4
	Integral boost 10%	0.964	0.745	0.718	3.82	0.349	160.8
	Integral boost 15%	0.963	0.714	0.687	3.64	0.350	191.3
	Integral boost 20%	0.958	0.700	0.670	3.52	0.354	228.0
	Integral boost 25%	0.960	0.683	0.655	3.38	0.355	255.3
Prescrip. func.	Polynomial, exp. 0.5	0.959	0.773	0.742	3.87	0.356	178.3
	Polynomial, exp. 1	0.962	0.753	0.724	3.74	0.354	187.3
	Polynomial, exp. 2	0.958	0.719	0.689	3.59	0.354	198.5
	Sigmoid, slope 2	0.963	0.703	0.677	3.57	0.349	194.3
	Sigmoid, slope 4	0.962	0.697	0.670	3.54	0.348	204.7
	Sigmoid, slope 10	0.966	0.695	0.671	3.53	0.348	200.6
Case	R-01-0.11 cc	0.970	0.839	0.815	3.75	0.361	86.3
	R-02-0.16 cc	0.937	0.701	0.656	4.30	0.271	102.9
	R-03-0.75 cc	0.947	0.815	0.772	2.88	0.451	229.2
	R-04-0.85 cc	0.952	0.910	0.867	2.88	0.517	186.8
	R-05-0.90 cc	0.936	0.673	0.630	3.44	0.329	153.8
	R-06-1.50 cc, 4 targets	0.985	0.667	0.657	5.15	0.223	139.8
	R-07-3.90 cc, 8 targets	0.962	0.463	0.445	5.72	0.151	410.4
	R-08-9.79 cc, 2 targets	0.978	0.763	0.747	2.97	0.405	152.8
	R-09-10.04 cc, 2 targets	0.984	0.713	0.702	2.89	0.403	177.3
	R-10-25.96 cc	0.965	0.687	0.663	2.45	0.407	300.3

*Abbreviations:* BOT = beam-on time; Cov = coverage; EI = efficiency index; GI = gradient index; PCI = Paddick conformity index; Sel = selectivity.

The values are the calculated means for each data subset, and the metrics are based on the mean prescriptions, which are fixed for integral boost prescriptions. BOT is calculated at 3 Gy/min. Mean metrics for the clinical single-prescription plans are provided for comparison. “*” indicates a modified metric to account for variable prescriptions.

### Isocenter generation comparison

Two cases, 1 irregularly shaped (R-05) and 1 with large volume (R-09), are used to explore automated isocenter generation, using the modified GSP algorithm and LGP fill algorithm to create sample treatment plans using more isocenters than present in the clinical plan. Each case uses dose painting prescriptions using a polynomial integral boost prescription function, with mean boost of 20%.

**Figure 3:**
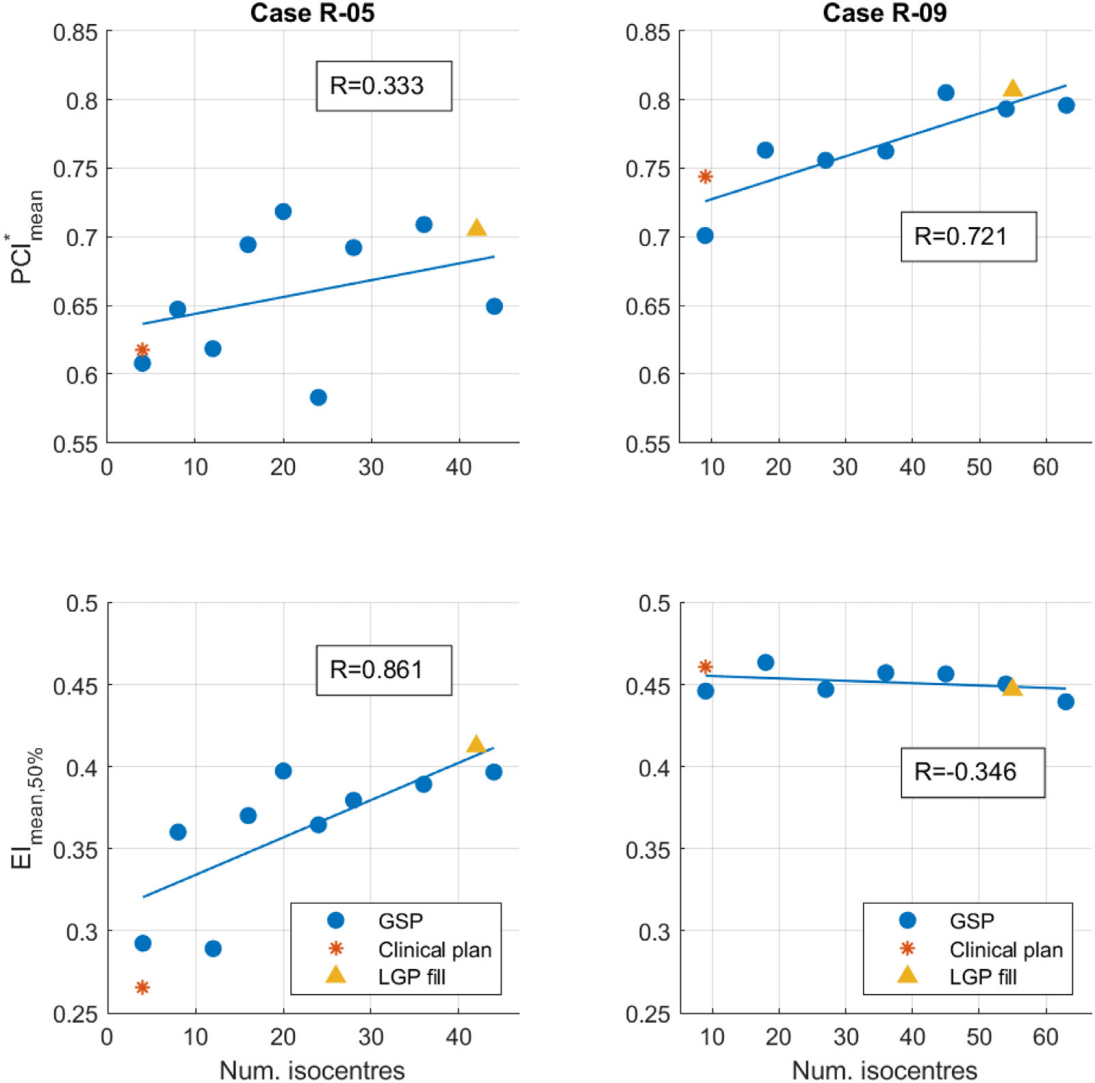
Plan assessment metrics for dose painting plans using automatically placed isocenters, plotted against the number of isocenters. Correlation coefficients for linear trend lines are displayed for plans with grassfire sphere-packing isocenters.

Figure 3 shows that, overall, there is a trend of improving plan quality as the number of isocenters increases. For case R-05, PCI^∗^_mean_ improves slightly, but improvement comes from normal tissue sparing as measured with EI_mean, 50%_ as shown with a linear trend line with correlation coefficient 0.721. For case R-09, EI_mean, 50%_ remains constant and PCI^∗^_mean_ shows a steady improvement, shown with a linear trend line with correlation coefficient 0.861. The numbers of isocenters placed with the LGP fill algorithm for these cases are high, and the plans score highly on both PCI^∗^_mean_ and EI_mean, 50%_, so existing clinical users with access to the tool already have a method to automatically place isocenters for dose painting.

### Computation time

**Figure 4:**
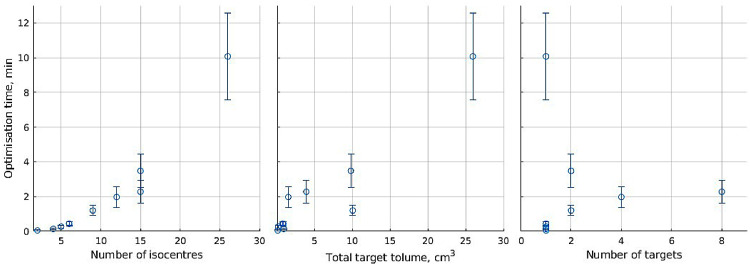
Dependence of optimization solve time on case features, averaged over all the dose painting plans generated for each case. Error bars indicate SD.

In Fig. 4, computational solve time for the optimized plans created in the section on dose painting plan quality is compared with measures of case complexity: number of isocenters, target volume, and number of targets. Number of isocenters has the strongest relation to solve time, followed by target volume, which is not unexpected given that the number of isocenters and the number of target volume voxels affect the number of variables and equations, respectively, in optimization calculations.

**Figure 5:**
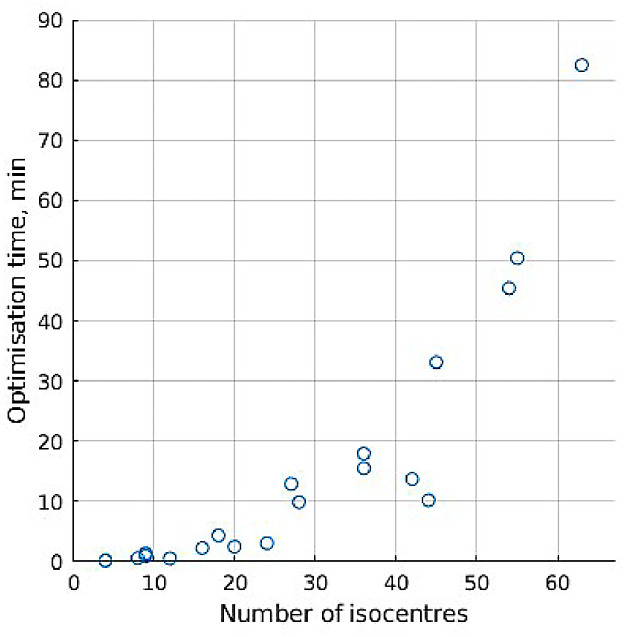
Optimization solve time for plans using differently placed isocenters.

For the optimized plans in the section on isocenter generation comparison, Fig. 5 shows agreement with Fig. 4, with number of isocenters having an exponential effect on solving time.

## Discussion

In this work, we investigated the plan quality achievable for dose painting in Gamma Knife radiosurgery. A variety of dose painting prescriptions were found, and optimization was applied to find treatment plans that delivered sufficient dose to targets while reducing dose elsewhere.

Overall, optimization is able to produce dose painting plans of reasonable quality, and the quality is affected by the choice of dose painting prescription method.

With the integral boost dose painting prescription method, choice of function form does not significantly affect plan quality much until mean boost exceeds 10%. Furthermore, dose painting plans using a polynomial prescription function with exponent 0.5 or 1 can use up to 25% mean boost with small decreases in assessment metrics compared with single prescription plans. The quantification of achievable dose painting plan quality here could inform subsequent clinical considerations of prescription function and boost amount, where the expected trade-offs in plan quality would be weighed against possible radiobiological considerations in dose painting, such as increasing dose to regions that require more dose (eg, lower radiosensitivity, high cell density) and vice versa.

An observation of the isodose lines in Fig. 2 shows that 100% to 140% are at some places close to each other, and the 140% isodose line covers a significant portion of some slices, which is not typical in clinical cases with single prescriptions. The explanation for this is that with dose painting plans, the isodose percentages are reflective of how we define 100%. In the figure, 100% was chosen as the minimum prescription, so a significant portion of the target would have upwards of 120% prescriptions relative to that minimum. With Fig. 2, a significant portion of the target is seen to have close to 140% of the minimum prescription. The isodose lines for these plans cannot be scaled by individual voxel prescriptions as they extend outside the target, which complicates percentage-based isodose analysis.

Nonetheless, there is an importance in avoiding overly high target doses, which leads to the section on isocenter generation comparison. The higher numbers of isocenters improve dose painting plan quality as degrees of freedom for dose delivery increase, and both the modified GSP algorithm and existing LGP fill algorithm are demonstrated as able to place these isocenters automatically. It is also possible to add to or adjust existing optimization penalties for overdosage. Further research may be done to refine isocenter placements, such as using dose painting prescriptions to guide placement.

As more isocenters are added, there may be a clinical concern of increased treatment time. A precise estimate is difficult without conducting physical measurements, but the BOT (radiation time) for our sample cases is mostly 100 minutes or more as seen in Table 1, which means the time from isocenter changes takes a smaller fraction of total treatment time. Further refinement of the optimization method, such as tuning the BOT penalty weight, will likely improve treatment time and may be the subject of further studies.

Optimization solve time for most plans using clinical isocenters is 5 minutes or less, which is well suited for clinical treatment planning even considering that a few iterations are usually done to fine-tune optimization weight settings. Using automatically placed isocenters, solve time remains short when using 24 or fewer isocenters, with the exact times dependent on solution algorithm and hardware used. Using more isocenters increases the degrees of freedom for dose delivery and potentially allow better plan quality in dose painting, so methods to shorten optimization time could be a direction for further study. Sampling methods to reduce voxels used, and thus the number of inequality equations in calculations, have been used successfully with single prescription Gamma Knife radiosurgery,^37^^,^^50^ which will improve solution time. On the other hand, as each voxel in dose painting has a unique prescription, sampling risks ignoring nonsampled voxels’ dose demands, so the exact impact of sampling on dose painting tumor coverage can be subsequently explored.

## Conclusion

Optimization for dose painting SRS is shown to produce treatment plans of reasonable quality, and the demonstrated trade-offs between plan quality of various prescription functions could help inform future clinical and research decisions on dose painting. Automated isocenter placement is shown to be compatible with dose painting treatment planning. As with current clinical treatment planning, plan quality improves through increased number of isocenters, but this needs to be balanced against computation times.

Further work could include planning studies to show indication-specific benefits when using dose painting or to consider alternative methods for isocenter placement beyond target geometry such as placing isocenters based on the dose painting prescriptions. Sampling could also be used to reduce optimization time, and its impact on dose painting plan quality may also be studied.

## Disclosures

Håkan Nordström and Nelly Nygren are employees of Elekta Instrument AB.
